# Obstacles to advancing women’s health in Mozambique: a qualitative investigation into the perspectives of policy makers

**DOI:** 10.1186/s41256-019-0119-x

**Published:** 2019-09-24

**Authors:** Mary Qiu, Talata Sawadogo-Lewis, Katia Ngale, Réka Maulide Cane, Amilcar Magaço, Timothy Roberton

**Affiliations:** 1 Johns Hopkins University – Bloomberg School of Public Health, Baltimore, USA; 2 Instituto Nacional de Saúde, Ministry of Health, Maputo, Mozambique

**Keywords:** Women’s health, Mozambique, Implementation, Policy making, Obstacles

## Abstract

**Background:**

Despite substantial investment in women’s health over the past two decades, and enthusiastic government support for MDG 5 and SDG 3, health indicators for women in Mozambique remain among the lowest in the world. Maternal mortality stayed constant from 2003 to 2011, with an MMR of 408; the estimated HIV prevalence for women of 15–24 years is over twice that for men; and only 12.1% of women are estimated to be using modern contraception. This study explores the perspectives of policy makers in the Mozambican health system and affiliates on the challenges that are preventing Mozambique from achieving greater gains in women’s health.

**Methods:**

We conducted in-depth interviews with 39 senior- and mid-level policy makers in the Ministry of Health and affiliated institutions (32 women, 7 men). Participants were sampled using a combination of systematic random sampling and snowball sampling. Participants were asked about their experiences formulating and implementing health policies and programs, what is needed to improve women’s health in Mozambique, and the barriers and opportunities to achieving such improvement.

**Results:**

Participants unanimously argued that women’s health is already sufficiently prioritized in national health policies and strategies in Mozambique; the problem, rather, is the implementation and execution of existing women’s health policies and programs. Participants raised challenges related to the policy making process itself, including an ever-changing, fragmented decision-making process, lack of long-term perspective, weak evaluation, and misalignment of programs across sectors. The disproportionate influence of donors was also mentioned, with lack of ownership, rapid transitions, and vertical programming limiting the scope for meaningful change. Finally, participants reported a disconnect between policy makers at the national level and realities on the ground, with poor dissemination of strategies, limited district resources, and poor consideration of local cultural contexts.

**Conclusions:**

To achieve meaningful gains in women’s health in Mozambique, more focus must be placed on resolving the bottleneck that is the implementation of existing policies. Barriers to implementation exist across multiple health systems components, therefore, solutions to address them must also reach across these multiple components. A holistic approach to strengthening the health system across multiple sectors and at multiple levels is needed.

**Supplementary information:**

**Supplementary information** accompanies this paper at (10.1186/s41256-019-0119-x).

## Background

The global health community has established that improving women’ health, in particular maternal health, continues to be a priority as we progress from the Millennium Development Goals (MDGs) to the Sustainable Development Goals (SDGs). Women’s health can be understood as uniquely important by applying a life course perspective. A woman’s health affects the health of her children, who in turn are affected as they progress through childhood, adolescence, and into adulthood [1]. Improving women’s health is imperative to improving population health more broadly.

In Mozambique, despite significant health gains in the past three decades, women’s health has continued to remain a challenge. Mozambique has an estimated maternal mortality ratio (MMR) of 408, placing it near the bottom of the global ranking for MMR [2]. Moreover, this number did not change from 2003 to 2011, according to the two latest DHS surveys conducted in those years [2, 3]. Estimated HIV prevalence for women between the ages of 15–24 is over twice as high as the prevalence rate for men [4], and only 12.1% of women are estimated to be using a modern form of contraception [3]. This is despite vocal commitment to improving women’s health at the highest levels of government. In a recent statement by the Government of Mozambique in July of 2016, the Mozambican president [5] announced his commitment to aligning his country with the SDGs, specifically emphasizing his commitment to the health of women, children, and adolescents.

Given this high level of political commitment alongside significant aid to the country from multi-lateral and bi-lateral donors [6], it is necessary to look at other factors that are impeding progress and limiting gains in women’s health in Mozambique. In recent years, public health practitioners have increasingly focused on implementation to better understand the performance of programs and policies. Existing evidence indicates that the delivery of evidence-based interventions and scaling up programs in resource poor settings has continued to be a major challenge. Many maternal health interventions have been proven effective, such as family planning and access to emergency obstetric care [7], yet the importance of implementation of maternal health programs has largely been overlooked across countries and programs [8]. Freedman et al. [8] highlight the urgent need for practitioners to shift their focus from the identification of strategies towards the implementation of those strategies. While policy formulation is important, the translation of policy into practice is equally important and can be complex in the context of local systems. Maternal health programs have, in particular, faced implementation challenges due to their reliance on external systemic factors such as availability of infrastructure, transportation, and socio-cultural norms amongst others.

There is a growing body of literature describing the nature of implementation barriers. Both Yamey and Puchalski Ritchie et al. provide frameworks to categorize the various types of barriers that have been identified in Low and Middle Income Countries (LMICs) to achieving successful implementation [9, 10]. Yamey breaks down implementation barriers into five levels: [1] attributes of the tool or service, [2] attributes of the implementers, [3] choice of scale-up approach or delivery strategy, [4] attributes of the “adopting” community, and [5] socio-political, fiscal and cultural context. Similarly, Puchalski Ritchie et al. identifies three key categories of barriers: [1] health system level barriers, [2] provider level barriers, and [3] patient/community level barriers. Within these three categories, the authors further identify 35 unique barriers, of which 31 were common to two country case studies or more.

In Mozambique, there has been limited work to understand the implementation process, and what bottlenecks exist. We conducted a study to assess the experiences of policy makers in the Mozambican health system to understand the process of formulating and implementing policies, and the challenges that have prevented Mozambique from achieving greater gains in women’s health. We present findings specific to implementation barriers in this paper.

## Methods

We undertook a qualitative study of policy makers in Maputo, Mozambique, from January 2017 to March 2017. The study team was a partnership between the Johns Hopkins School of Public Health (JHSPH) and the Instituto Nacional de Saúde (INS) in Mozambique. Ethical clearance was obtained from the JHSPH Institutional Review Board and from the Institutional Committee of Bioethics at INS.

### Sampling

We conducted sampling in two phases: [1] systematic random sampling of an initial sample of 20 individuals; and [2] snowball sampling to identify additional participants.

From a list of staff of the Mozambique Ministry of Health (MISAU), we identified 95 individuals whose department was deemed relevant to women’s health issues, whose role was deemed relevant to the policy-making process based on job title, and whose position was as senior as Department Chief or higher. Of the 95 identified individuals, 59 were women and 36 were men.

We used systematic random sampling to select a starting sample of 15 women and 5 men. We oversampled female participants because we were interested in exploring the experiences of female policy makers in the policy-making process. We used Excel to conduct systematic random sampling, creating two lists of women and men, using the RAND function to order women and men randomly, and selecting every 4th woman and 7th man to further ensure randomization.

At the end of each interview, we asked participants to suggest three additional participants who they thought would be important to include in our study. Every person that was recommended in this way was added to the sampling list. If the person had not already participated in the study, he or she was contacted by the study team and asked to be interviewed. Such snowball sampling was continued until the study team concluded that saturation had been reached and there was no need to interview more participants.

In total, 39 participants were interviewed, of whom 32 were female and 7 were male. See Table 1 for additional details on participants. Most participants were senior level staff with over 5 years of policy-making experience.

**Table 1:** Participant characteristics

		Gender	
		Female (*n* = 32)	Male (*n* = 7)
Years of Experience	0–2	0	1
	3–5	2	0
	5–10	7	2
	10–20	11	3
	20+	11	1
	Unknown	1	0
Self-identify as policy maker	Yes	18	2
	No	14	5
Institutional affiliation	Ministry of Health	27	5
	NGOs	4	1
	Municipality of Maputo	1	1

### Data collection

We recruited three Mozambican data collectors with experience conducting qualitative research and held a 2-day training on methodology and to familiarize data collectors with the interview guide.

Data collection took place over a period of 3 months, from January to March, 2017. All interviews were conducted in Portuguese, at a location and time that was convenient to the participant. Data collectors scheduled appointments with participants by phone where possible or attempted to schedule appointments in-person at the INS when contact information was not available. Data collectors made 3 attempts to reach each participant, before the participant was removed from the sampling list and replaced. The next participant on the list was then interviewed until the list was exhausted. After this, participants were selected from the list of individuals recommended by the original participants.

Oral and written consent were obtained from each participant before the interview was administered. Interviews were recorded using an electronic voice recorder, and data collectors took detailed notes throughout the interview. Data collectors used a semi-structured interview guide developed by the study team, that focused on both the experience of the policy maker in their respective role, and what they personally felt were priorities around women’s health in Mozambique. Each interview lasted on average between 30 to 45 min. The interview guide can be found in Additional file 1.

We continued this process until reaching saturation, at 39 participants, when we felt that new themes were no longer being offered or elicited from participants.

### Data analysis

Data collectors uploaded the interview recordings onto study laptops after each day. Interviews were transcribed verbatim in Portuguese, and data collectors produced short summary reports of each interview. The study team met regularly to discuss emerging themes and to triangulate findings across participants.

We used a combined deductive and inductive method to develop a codebook, with some codes determined from initial reviews of available literature, and some as they emerged from the data through regular debriefing meetings. Transcripts were uploaded into Dedoose (version 7.6.6), and the study team developed a preliminary codebook by identifying major themes using 8 (20% of total transcripts) randomly selected transcripts. Our initial review of literature also guided this step.

We then used this first codebook to again code 8 randomly selected transcripts – excluding the initial 8 from the selection. After making some final adjustments to the final codebook based on our findings in this second round of coding, we then proceeded to code all transcripts in Portuguese.

The coded excerpts were exported from Dedoose into Excel and were then re-arranged by themes. Key themes presented in our results section below were identified on the basis of the frequency at which participants discussed them. Important excerpts were selected for inclusion in this paper.

## Results

There was a consensus amongst participants that while maternal and child health is currently being prioritized by the government, implementation processes are inadequate and pose a barrier to improving outcomes in the country.

“*We keep saying that [maternal and child health] isn’t being prioritized, that they aren’t being prioritized… But I think no. They’re definitely being prioritized, but the difficulty – the big barrier – is the implementation.”* – Participant 2.

*“I think that’s not a problem of policies because in many places you’ll hear people talking about how it’s a priority, but to make it real we need to do some things, we need to implement. That’s where the weaknesses are.”* – Participant 4.

Throughout interviews, participants discussed various barriers that they had either faced or witnessed in the implementation of maternal and child health policies. These barriers typically fell into one of three categories: [1] obstacles that occur during the policy making process itself, [2] difficulties due to external funding, and [3] the disconnect between the policy making arena and the realities on the ground.

### Barriers to implementation within the policy making process

#### Lack of systematic approach

Participants described a situation where they face emergency situations one after the other, without a systematic approach to the development and implementation of policies. They describe feeling pressured to deal with emerging issues, “*attacking public health issues as if we were firefighters*”, as Participant 25 put it. Participants experienced particular difficulty when juggling competing priorities.

*“The national evaluation says that we’re not doing well in this health facility so we need to flip around and attack that indicator, without forgetting that we have those other indicators which are also priorities, but there are priorities within priorities… So we have to do these gymnastics to see where to invest time and money without forgetting about any indicator. These evaluations are every three months.”* – Participant 25.

Participants attributed a portion of the perceived inefficiency in implementation to this phenomenon. Some viewed their role as being responsive rather than proactive, with abrupt changes at the policy-making level creating obstacles to implementation. Each new policy change has repercussions in terms of dissemination of the policy itself, development of implementation plans, and potential new human resources or infrastructural needs. Frequent modifications of the policies do not allow for downstream levels to adjust to changes before they are once again modified, creating confusion at all levels.

*“When we’re already more or less good, we change the policy! We change it, and we always stay in this process of ‘implement, implement’. Sometimes, [staff] finds it difficult to adapt immediately to new policies, to new things” – Participant 22.*

A recommendation that was brought up by multiple participants was to put greater emphasis on monitoring and evaluation (M&E). Some participants felt that M&E was simply not being done, although some said that there were M&E components to most policies.

“*We’re improving, but the monitoring and evaluation is still a big challenge. Measuring what we’re doing and creating reports that can show us the way forward, the progress and where we need to do better... So I think we need to increase this monitoring component which is important”* – Participant 6.

*“Our big problem is monitoring. By the time we realize [we need to do it], a lot of time has already passed and it’s late, so we end up not doing it.”* – Participant 26.

#### Lack of monitoring and evaluation

Many participants highlighted the lack of M&E as a barrier to the policy making process. Along with the rushed pace of policy making, participants flagged that since priorities shift often, some activities are abandoned or paused mid-way. By the time they’re evaluated, no significant change has occurred. Without assessing the implementation of a program, it is difficult to judge whether the program was inefficient, or it simply was not implemented well.

*“This is where we fail. We have good policies, very well designed, with implementation, monitoring. But if I start with one activity and there’s no evaluation at all, after a while I get around to evaluating it and see it failed. I abandon it. So there isn’t that feedback loop.* [...] *Things aren’t dealt with on a deeper level. We don’t invest in fixing the problem”* – Participant 25.

*“One of the major constraints that we have is that we have so many plans, so many activities that we need to guarantee that they’re implemented. Now the tension is: in all of these, which ones are those that will really give results?”* – Participant 25.

#### Misalignment and integration of policies across sectors

Around policy implementation, one issue that was frequently mentioned was that of aligning policies with each other and integrating policies across sectors. Participants felt that improving women’s health was not solely the purview of the Ministry of Health. For example, one policy maker cited the example of breastfeeding. While the official recommendation from the Ministry of Health is that women breastfeed for 6 months, women are only allowed 2 months of maternity leave.

*“In the Ministry of Health we said that the woman has to give exclusive breastfeeding until 6 months, but Labor Laws only give a maternity leave of two months – how is a woman going to do that? … we need to have policies that complement each other.”* – Participant 22.

Other ministries also play a role in ensuring that women have access to the health services they need and the infrastructure necessary for them to achieve better health. However, multi-sector cooperation was described as being unsuccessful to date. Unified action across Ministries is recognized as being necessary, but not achieved.

*“I’m designing a maternal health strategy. What does [the ministry of] Social Action need to do? Culture? […] Public Works needs to guarantee that there’s a road […], Transport also […]. After this, us as Health we can do that one part of it”* – Participant 3.

*“If I don’t have a road, how can I bring women to health facilities? If I don’t have water, how can I tell mothers that they must wash their food with boiled water to reduce diarrhea deaths? [...] If we don’t have these conditions, how can I ask her to follow our policy?”* – Participant 17.

#### Lack of capacity of policy makers and implementers

According to participants, many individuals who work in the ministry of health are medical professionals such as doctors and nurses, and do not have experience in policy making or implementation. As participant 26 put it: “*we’ve got colleagues who have a lot of schooling and lots of knowledge, but when we try to translate that knowledge into practical work, it gets difficult. We’ve got people with degrees from prestigious schools but who struggle to implement the theory into practice*”.

Lack of personal motivation, lack of motivating factors, lack of mentorship, and lack of training were all flagged as reasons for a perceived low technical capacity at the policy making level. However, of these, lack of training was most often flagged. Participant 5, a female senior implementer painted this picture of her first experiences in an implementation role: “*One quick week of rotation in medical school on programs and all of a sudden I had to do project management at the health facility level* […] *because I was director of that health facility. What does that even mean? I didn’t know what that was”.* Other participants echoed similar situations of either feeling insufficiently prepared for policy making or implementation roles, or of only receiving training on an ad hoc basis.

### Difficulties due to external funding

#### Donors determine what is and is not done

Donors are seen as playing a big role in the planning and implementation of health policies in Mozambique. Participant 2, a senior female policy maker described the problem with this: “*we live off donations, but that’s not how to do it. It’s the system. We need to strengthen the health system. We keep talking about it, but what investment is made in this health system strengthening?”.* Participant 23 put it as: “*Unfortunately – yes, that’s exactly right – unfortunately, this is the situation because our country needs funds to implement health*”. Participants see donors as crucial players because they provide the resources necessary for policy implementation, but this support is tied to the donor’s agenda instead of what participants perceive to be the country’s own priorities.

*“The correct term is really ‘impose’. I say ‘impose’ but we who work in ‘cooperation’ try not to use that term... But it’s exactly that, they ‘impose’. It’s arrogant. They really impose. They allocate the resources, and then they say ‘do this, do that’. If I say ‘I need to fight diarrhea, this is our big issue’, they answer ‘for me, diarrhea isn’t a priority’”* – Participant 30.

Donors are come from outside of Mozambique, and many participants felt, as such, some donors were not interested in doing what is best for Mozambique, but rather follow their own organization’s agenda. Participant 30 said: “*It’s got a lot to do with the availability of funds and whatever’s trending in whoever has money’s preoccupations. Whoever has the money, gets the say-so*”. Some participants were saddened that they were not able to fund local priorities, with participant 37 saying: “*it creates a feeling of impotence*”.

#### Vertical programming as a result of the donor system

Donor-influenced policies were described as being too “vertical”, with little to no funding available for strengthening the health system itself. Participant 14, a female senior policy maker, said: “*we’re not going to invent things. Our focus is strengthening the health system in strategic areas. It’s clearly identified: for us to improve mother and child health [the Ministry of Health] needs to improve [health] in the country in general*”. This vertical approach to programming means the government is not able to fund the health system holistically. For example, the HIV and AIDS initiatives were perceived as being a particularly well-funded, to the detriment of other initiatives.

“*Some partners come with a pre-determined area to finance [...] they only want to finance malaria, or only HIV. But we’re a system. All of this ends up in one single health facility, which is where you have individuals, so it’s very difficult to separate all of these things out*” – Participant 6.

*“Sure, the woman will do her PMTCT treatment [for HIV], but she’ll die from eclampsia” –* Participant 23.

This complex funding landscape makes long-term planning difficult. Changes in donor priorities do not allow programs to achieve impact before they are changed, and create barriers to sustainability.

*“They need to be sustainable policies, but countries suffer from lot of [external] pressure. If they don’t accept certain policies, they won’t have financing to continue. This is why you see these constant changes in the HIV policy, which is not good because you need to cement certain things.”* – Participant 2.

### Disconnect between policy and implementation

#### Poor dissemination of policies

Many participants suggested that policies are poorly disseminated at the level that change is intended to take place. Participant 5, a female senior policy maker, sees it as: “*when we approve a policy or a strategy, it often just stays at the central level. It’s not disseminated down to the health facility level*”. This was viewed as a significant barrier to implementation.

*“I think it’s the appropriation and dissemination, because if you don’t know about it, obviously you won’t do it. When the policy is made, it’s not for the Ministry of Health to use. It’s for all the providers, whether they’re in the private sector, an NGO, in the general public… Everybody should follow it, but if you don’t make sure the information reaches the base, it’s clear that it will never work. That’s the biggest challenge”* – Participant 16.

Participants described a communication breakdown between national, provincial, district, and local levels. Participant 5, a senior female implementer, gave this example: “*Oftentimes, we at the provincial level, we learned of policies or strategies from television! Like… ‘what’s that?’[...]. Now we’ve started to have more interaction [with the central level]*”. While most participants did not offer such striking examples, many agreed that policy dissemination to provincial, district and health-facility level was lacking.

“*First, the difficulty is to get people to understand what we’re trying to do […]. Another difficulty we have [...] is translating what is written in the document into practice. We’re a Portuguese language country and others are English-speaking. All the documentation comes in English and that really creates a big difficulty for us to first translate and then implement in our sector*” – Participant 5.

However, participants did view radio or television – especially in local languages – as important methods to communicate health messages to the public. Participant 28, a male junior participant, saw these communication media as particularly appropriate, because: “*the first challenge is access to information. Access to information is something I see as fundamental especially since a big part of the Mozambican population is female and illiterate*”.

#### Financial and human resources

In some participants’ opinions, policy makers do not take the financial and human resource limitations at the district level into account, and therefore impose unrealistic targets and expectations.

“*It’s interesting to see when we’re in these meetings talking amongst directors, among our colleagues. When some proposals are made without taking into account the realities of the base level, those of us who have actually worked at the base level look at each other and say ‘yeah… that’s never going to work’”* – Participant 5.

Indeed, participants felt that limited resources severely impact the implementation of policies and their impact. Participant 38, a senior policy maker, said: *“we want to implement but we get stuck because we don’t have resources to implement, so the policies stay there, stored away. [...] at the end, we’ll do some monitoring and find that nothing happened because there were no resources for implementation”*.

#### Cultural context

Another theme that emerged was the need to better engage men in improving women’s health, and to consider them in the design of new policies. Participants noted that in Mozambican culture, men are the household decision makers and often determine various aspects of a woman’s health. For example, men may decide whether and when a woman has a child, or whether she seeks health services. The issue of women’s autonomy or lack thereof is often overlooked, and while services or programs may exist, the ability of women to access these services may be limited.

*“I think in general, we can’t say today that women aren’t prioritized. What’s happening is that the men are really being left behind and afterwards these [men] are the people who make decisions, without understanding what’s necessary. [The man] will always affect directly or indirectly women’s and children’s health, so we have to think about it differently. […] we forget the cultural aspects, in which the man is also part of his family. He’s the decision maker.”* – Participant 2.

#### Need for centralized coordination but decentralized action

District-level staff are sometimes instructed to implement a policy that does not address their own district’s priorities. If the people on the ground – those carrying out the policies and the beneficiaries themselves – do not identify with a policy, the implementation of that policy will suffer.

*“We cannot ever make a policy […] here, at the central level, and then we go and apply it to an area where people don’t identify with it. That’s a total failure.”* – Participant 13.

*“An intervention can be applicable in one context and not in another. [there’s a need to] evaluate the acceptability of this strategy in the location where we plan to implement but we don’t manage to do that; this [policy] will be implemented from North to South”* – Participant 1.

Despite these issues, participants recognized that the Ministry of Health does have an important role to play to coordinate efforts in the health sector overall. Participant 5 acknowledged: “*we need to make sure efforts are coordinated. For example, we have the government that has a budget, the NGOs have their budgets… Some give support to the Ministry of Health, others go directly to the communities. They do what they want, when they want. [some donors] give to other NGOs, others give to local NGOs. We need the government to coordinate all of this*”.

Participants generally viewed this centralized coordination in a positive light, as an appropriate and important function of the Ministry of Health. However, most emphasized the need for flexibility so that policies can be contextualized at the sub-national level.

*“It’s true that the initiative to create a policy occurs at the central level – as it should, because that’s what the ministry [of Health] is for: to give technical orientation. But for a while now we’ve realized we also need to bring the conversation down to the provincial level. That would be optimal, based on what we’re seeing in the women and child health field”* – Participant 5.

## Discussion

The findings from our study corroborate the existing literature around barriers to implementation of women’s health programs. Policy makers in Mozambique elucidated three key categories of barriers: [1] obstacles that occur during the policy making process itself, [2] difficulties due to external funding, and [3] the disconnect between the policy making arena and the realities on the ground. Within these three categories, we identify nine specific barriers, of which eight are included in existing frameworks [9, 10].

While the nature of factors discussed by policy makers ranged in topic and the level at which they occur, they can be linked to a higher-level need for health systems strengthening in Mozambique, and improved coordination in policy making and implementation, both across sectors and levels of government. Connecting implementation barriers to well-known health systems components, we can see that the majority of the factors described are not specific to women’s health, but applicable across a range of health issues.

### Leadership and governance

The WHO describes governance as “the exercise of political, economic, and administrative authority in the management of a country’s affairs at all levels, comprising the complex mechanisms, processes, relationship and institutions through which citizens and groups articulate their interests, exercise their rights and obligations and mediate their differences”. This concept of governance can be linked to a number of challenges described by policy makers in our study, including the capacity of policy makers, the need for multi-sectoral policies, and donor influence.

Specific to strengthening the capacity of policy makers, there is a growing body of evidence indicating the need to support the capacity building of policy makers to both digest evidence, and translate evidence into policy and practice [11, 12]. This is particularly relevant to maternal health policies given the large number of proven interventions, and a plethora of combinations in which single interventions can be delivered as packages of care [13, 14]. In a review by Clar et al. [11], common factors found to facilitate the knowledge translation process included successful collaboration with and involvement of all stakeholders, strong leadership, and targeted training to policy makers. These factors reflect the comments by policy makers in our study on the lack of preparation and training they received before assuming their roles. In Pakistan, a review of maternal and child health policies noted similar findings, with institutional capacity being necessary to translating policies into actual service delivery [15].

Our participants also described a chaotic policy-making process, with frequent changes to policies and poor dissemination of information to districts and health facilities. In Pakistan, similar observations were noted. Authors felt that this sent inappropriate and confusing signals to health managers and providers, stemming from distrust in past governments, which subsequently weakened the implementation process [15]. In Uganda, rapid changes to user fee policies resulted in facility level drug shortages and health workforce shortages [16]. High-level policy makers in Mozambique should adopt a more deliberate process when implementing or changing existing policies, to avoid unintended negative consequences as changes are felt at each level of government.

Many participants discussed what they saw as an excessive – and to some degree detrimental— degree of control held by donors in shaping health policies and programs in Mozambique. This finding is echoed by Yamey [9], who describes how poor coordination between donors may impede implementation. In an analysis by Khan et al. [17] on donor influences in Cambodia and Pakistan, they noted that donors exercised power in three different ways, including through the control of knowledge and evidence. It is possible that strengthening policy makers capacity to engage with evidence and information in the broader policy making and implementation process may also influence their ability to engage donors and strategically align donor priorities with country level priorities [17].

In regard to aligning policies across sectors, there is increasing recognition for the need to adopt a multi-sectoral approach to address complex health issues. This is evident in the SDGs, in which many of the goals listed involve, or affect, sectors outside of health [18]. To achieve long-term goals, policy makers should work across sectors to formulate policies that complement each other. To this end, increasing the capacity of policy makers should include supporting multi-sectoral engagement and collaborative relationships for the creation of more comprehensive and holistic policies.

### Health Care Financing & Health Workforce

Participants discussed the lack of financial and human resources available to implement policies, particularly at the sub-national level. Resource mobilization has been highlighted previously by Yamey [9] and Puchalski Ritchie [10] as barriers to implementation. While Mozambique continues to rely on external sources to fund health programs, opportunities for domestic resource mobilization may exist through increasing efficiencies in the health system. Recent discoveries of natural resources in the country may also provide future revenue for the health sector, if these resources are managed effectively [19]. Historically, there has been limited taxation of large natural resource projects in the country [19].

Participants also highlighted the challenges around the Mozambique health workforce, both at national and service-delivery level. Specific to health workers, Mozambique continues to suffer from a dire health workforce shortage, particularly in rural areas [4]. Poor working conditions and low pay have resulted in the ‘brain drain’ of trained health workers, both overseas and to the private sector within the country. Increasing resource allocation to health workers, increasing the number of health workers that are trained each year, and shifting tasks to lay health workers are all strategies that could be used to address this challenge [20].

### Health information systems

Strong M&E systems are critical to the success of programs and policies [10]. Assessing the implementation of programs, particularly at the process or output level, enables learning that can and should be used to then refine programs over time [21]. To achieve strong M&E systems, countries need robust health information systems infrastructure and accurate and timely data. In Mozambique, disparate implementation of information systems supported by a variety of donors has resulted in a fragmented system, with variability in consistency of data entry and management [22]. Although national level information systems have been implemented (such as SISMA, eSip-Saude), there is limited information on the quality of this data and how well this system is functioning. Further efforts to strengthen information systems and streamline reporting across platforms and donors could provide policy makers with necessary information on implementation strength, and reveal components of programs or policies that need improvement.

#### Limitations

Our study used qualitative data to elucidate insights into the policy implementation process in Mozambique. Qualitative data presents individual level perceptions, which are influenced by internal biases and experiences. While our team used established data analysis techniques, there are still a number of limitations to our study. First, policy makers may have been reluctant to express their full opinion with our study team, given the nature of their positions within a government institution. Second, we did not interview policy makers outside of the National Ministry of Health. As such, views on implementation at local levels were not corroborated by participants directly working at that level.

## Conclusions

Women’s health in Mozambique continues to face barriers, despite political commitment at the national level. Our study highlights implementation as a critical bottleneck to effectively translating policies at the national level to service delivery at local levels. Participants raised challenges related to the policy making process itself, the disproportionate influence of donors, and the disconnect between policy makers at the national level and realities on the ground. Each of these factors can be seen through a health systems lens, with the implementation issues – and their potential solutions – touching multiple health systems components. While policy makers spoke to the specific challenges surrounding the implementation of women’s health programs, we believe their comments are relevant to other health areas, in Mozambique and elsewhere. Improving women’s health will require a holistic, multi-sector strategy that goes beyond individual programs, requiring a continued commitment to strengthening the health system in its broadest sense.

## Supplementary information

**Additional file 1:**. Interview guide used to guide the individual in-depth interviews with participants in the study. (DOCX 23 kb)

## Data Availability

Please contact the corresponding author for data requests.

## Abbreviations

INS: Instituto Nacional de Saúde (National Health Institute - Mozambique)
JHSPH: Johns Hopkins School of Public Health
LMIC: Low and Middle Income Countries
M&E: Monitoring and Evaluation
MDG: Millennium Development Goals
MISAU: Ministério da Saúde (Ministry of Health - Mozambique)
MMR: Maternal Mortality Ratio
NGO: Non Governmental Organization
SDG: Sustainable Development Goals
WHO: World Health Organization
